# Heart surgery in a pediatric patient with congenital heart disease and hemophilia B: A case report from Sudan

**DOI:** 10.1002/ccr3.7901

**Published:** 2023-09-10

**Authors:** Mohmmed A. Mohmmedahmed, Esra M. Eltahir, Amna M. Mohammedali, Bashir A. Yousef

**Affiliations:** ^1^ Pediatric Cardiac Intensive Care Department Sudan Heart Centre Khartoum Sudan; ^2^ Pediatric Cardiology Department Sudan Heart Centre Khartoum Sudan; ^3^ Faculty of Pharmacy University of Khartoum Khartoum Sudan

**Keywords:** congenital heart disease, hemophilia B, Sudan, ventricular septal defects

## Abstract

**Key Clinical Message:**

A multidisciplinary team approach, careful hemostasis, and factor replacement therapy are important in the perioperative management of hemophiliac patients undergoing pediatric cardiac surgery.

**Abstract:**

The combination of congenital heart diseases (CHD) and hemophilia is rare; furthermore, heart surgery and perioperative management of such cases is challenging. This report illustrates the challenges of pediatric cardiac surgery in an infant with both hemophilia B and CHD. Multidisciplinary team approach, careful hemostasis, and factor replacement therapy were key to success without hemorrhagic complications before, during and after surgery.

## Graphical abstract

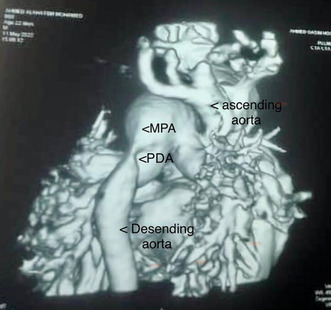

## 1 INTRODUCTION

The incidence of congenital heart disease (CHD) is approximately 8 in 1000 births while the incidence of hemophilia A is around 1 in 5000 male birth and hemophilia B is around 1 in 30,000 male births.^1^, ^2^

An association of CHD and hereditary bleeding disorders like hemophilia is uncommon, the incidence of CHD in hemophilic patients is almost the same as the incidence of CHD in general population.^3^ Although few pediatric cases have been reported with this unusual combination have successfully undergone heart surgery.^4^, ^5^, ^6^, ^7^, ^8^ The literature reported few pediatric cases underwent successful heart surgeries with this unusual association. 4–8 and up to our knowledge, a combination of interrupted aortic arch (IAA) and hemophilia B was not reported. We aim to emphasize in this case report the importance of the multidisciplinary team approach in the perioperative management of a child with hemophilia B and type A (IAA) with ventricular septal defects (VSDs) in our cardiac centre in Sudan.

## 2 CASE REPORT

A term boy was born to non‐consanguineous parents after an uneventful pregnancy through spontaneous vaginal delivery in the hospital. He was sent home 4 h after birth and readmitted after 3 days with shortness of breath and poor feeding. There was no significant family history of CHD or hematological diseases; the patient was tachypneic and distressed; he required oxygen; and the systemic examination was normal. Four limbs' blood pressure and upper and lower limbs' oxygen saturation (SPO_2_) measurements were not recorded in the peripheral hospital due to a deficiency of required equipment.

**FIGURE 1:**
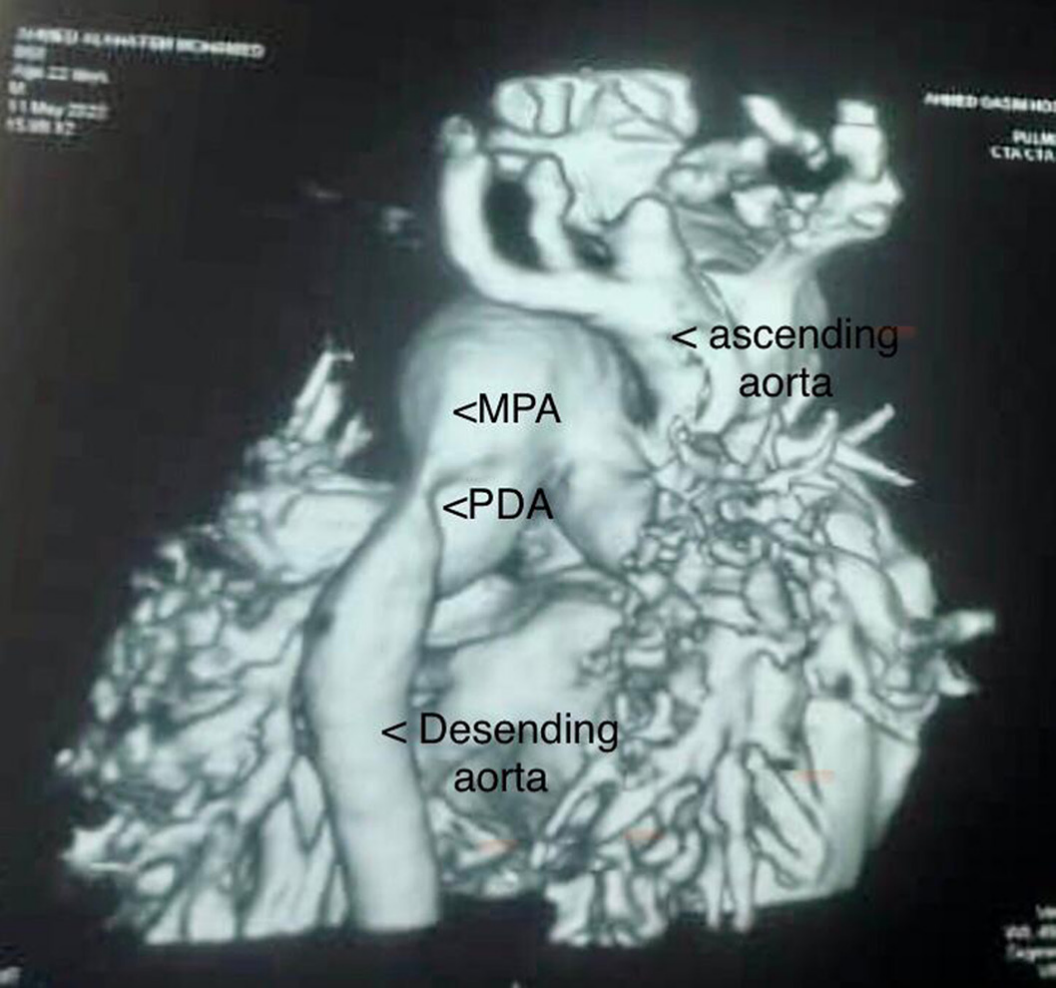
Computerized tomography angiogram demonstrated the diagnosis of interrupted aortic arch type A (just past the left subclavian artery), and the descending aorta supplied by patent ductus arteriosus.

The patient was initially diagnosed with early neonatal sepsis due to high C‐reactive protein (CRP) and total white blood cell (WBC) count and was given empirical antibiotics for 10 days with no significant improvement in symptoms. Further investigation showed mild cardiomegaly in chest X‐ray (CXR), but the cardiology evaluation was delayed due to lack of pediatric cardiology services in the hospital. The diagnosis of type A (IAA), patent ductus arteriosus (PDA), and multiple (VSDs) was confirmed on day 19th of life based on echocardiography and cardiac CT angiography (Figure 1).

During the course of admission, there was a serious suspicion of bleeding tendency diseases, manifested by repeated bleeding from puncture sites. Initially, the patient was treated as disseminated intravascular coagulopathy (DIC) in relation to sepsis and frequently received fresh frozen plasma (FFP) and vitamin K. The patient was referred to the tertiary pediatric intensive care unit (PICU) for further evaluation and management. Hematological workup under hematology team supervision confirmed the diagnosis of hemophilia B based on the following results: high APTT, normal PT, normal bleeding time, and factor assay were as follows: normal VWF: 121% (45–140), normal factor VIII level: 150% (65–140), and low factor IX level: 44% (60–140). Hemophilia center recommended not to give FFP before surgery with care on sampling, handling, and avoiding intramuscular injections.

The infant was sent to the tertiary cardiac center on day 45 of life after the resolution of sepsis for cardiac surgery with a detailed hematology plan from hemophilia center to prevent bleeding complications during and after the surgery. He underwent (aortic arch repair + pulmonary artery (PA) banding and PDA ligation).

The patient received 100 IU/kg of factor IX during induction of anesthesia in order to keep the factor level around 100%, followed by 50 IU/kg every 12 h for 48 h and then 50 IU/kg every 24 h for 2 days. According to the hematology team plan, there was no need for postoperative repeated factor IX level as far there was no bleeding complication and acceptable coagulation profile and APTT levels (Table 1). The surgery was successful without major complications, with the exception of postoperative transient systemic hypertension which was managed according to PICU protocol. The patient was extubated to CPAP after 3 h postoperation and weaned off CPAP after 9 h to nasal cannula then to room air by day 2 of surgery. His total ICU stay was 5 days; all catheters and drains were removed routinely without any concern; he was discharged from the hospital after 7 days in a stable condition; and all investigations including coagulation profiles were normal.

**TABLE 1:** Hematological investigations and interventions during the admission before and after cardiac surgery and in follow‐up.

Date	Platelets count (cells/μL)	PTT (s)	PT (s)	INR	Factor IX level (units/dL)	Factor IX therapy	Adjunct therapy	Remarks
Day 1 of admission in general PICU	235 × 10^3^	62	16	1.6			Receive fresh frozen plasma (FFP)	Suspicion of DIC secondary to sepsis receive FFP
Day 4 of admission in general PICU	248 × 10^3^	35	12	1.3	44			Factor XIII and VWF were normal diagnosed as hemophilia B
Intraoperative (cardiac surgery)	257 × 10^3^	83	22	1.4		100 IU/Kg STAT	Receive tranexamic acid and FFP	No major bleeding during or after surgery
Day 0 post op in cardiac PICU	250 × 10^3^	40	18	1.5		50 IU/kg BID		
Day1 post op in cardiac PICU	246 × 10^3^	61	25	1.6		50 IU/Kg BID		
Day 2 post op in cardiac PICU	220 × 10^3^	89	20	1.1		50 IU/Kg OD		All invasive lines, catheters, and chest drains were removed without complications
Day 3 post op in cardiac PICU	236 × 10^3^	40	21	1.3		50 IU/Kg OD		
Day 7 post op (discharge day)	175 × 10^3^	49	16	1				
14 days after discharge in hematologic clinic					23			
30 days after discharge (in hematologic clinic)		51	12		7.5			

Abbreviations: BID, bis in die, twice a day; DIC, disseminated intravascular coagulation; INR, international normalized ratio; IU, international units; Kg, kilograms; OD, Once a Day; PICU, pediatric intensive care unit; PT, prothrombin time; PTT, partial thromboplastin time.

The patient is under regular follow‐up with pediatric cardiology and hematology, with normal growth and development for his age, and planned to have a complete repair (PA de‐banding and VSD closure) by the age of 6 months.

## 3 DISCUSSION

An association of CHD and hereditary bleeding disorders like hemophilia is relatively rare and uncommon. Jedele and his research team demonstrate that the frequency of CHD in hemophilic patients is 0.75% with no major difference from the frequency of CHDs in the general population which was around 0.8%.^3^

Few pediatric case reports have shown a combination of transposition of the great arteries (TAG) and hemophilia that have successfully undergone corrective surgery using a multidisciplinary team approach and strict hematology plans.^8^ Furthermore, three reports demonstrated perioperative challenges in cardiac surgery in hemophilic patients with tetralogy of Fallot (TOF).^4^, ^5^, ^7^ Other reports demonstrate successful surgeries of total anomalies pulmonary venous connections (TAPVC) and successful coarctation of aorta (CoA) repair in patients with hemophilia A.^6^, ^9^ On the other hand, the combination of type A (IAA) with multiple VSDs and hemophilia B was not reported in the literature.

Pediatric cardiac surgery in countries with limited resources such as Sudan is challenging due to crucial deficiency in specialized medical personnel and lacking resources.^10^, ^11^, ^12^ Tackling complex cases such as our case in this report in our limited setup is quite difficult and challenging.

Good collaboration between all pediatric disciplines (cardiology, neonatology, intensive care, hematology, cardiac anesthesia, and cardiac surgery) was the cornerstone of our success in our deficient and underdeveloped configuration in our center. We used local Sudan hemophilia guidelines which were developed from updated WFH guidelines for the management of hemophilia.^13^ We followed factor replacement therapy for major operations using factor IX clotting factor concentrate either plasma‐derived or recombinant targeting plasma factor IX level between 60 and 80 IU before the surgery and keeping the level between 50 and 70 IU postoperatively up to 14 days if needed (Table 1).

The literature demonstrates structural and organized protocols for hemophilic patients undergoing cardiac surgery with perioperative, intraoperative (before, during, and after bypass), and postoperative considerations. Factor replacement therapy and factor level monitoring perioperatively with good surgical and hemostasis techniques and the use of adjunct pharmacological agents, such as tranexamic acid, epsilon aminocaproic acid, and desmopressin were described in these protocols.^14^, ^15^

## 4 CONCLUSION

Careful hemostasis and factor replacement therapy protocol using a multidisciplinary team approach are crucial for the management of hemophilia patients undergoing pediatric cardiac surgery.

## AUTHOR CONTRIBUTIONS

**Mohmmed A. Mohmmedahmed:** Conceptualization; data curation; formal analysis; investigation; methodology; supervision; visualization; writing – original draft; writing – review and editing. **Esra M. Eltahir:** Data curation; investigation; methodology; writing – original draft. **Amna M. Mohammedali:** Data curation; formal analysis; investigation; methodology; writing – original draft. **Bashir A. Yousef:** Conceptualization; resources; visualization; writing – original draft; writing – review and editing.

## FUNDING INFORMATION

None.

## CONFLICT OF INTEREST STATEMENT

The authors declare no competing interests.

## ETHICS STATEMENT

No ethical approval is needed for this type of publication in our institution. However, the patient's parents have given consent to this publication.

## CONSENT

Written informed consent was obtained from the patient's parent for publication of this case report and any accompanying results and images. A copy of the written consent is available for the review by the Editor‐in‐Chief of this journal on request. Permission to reproduce material from other sources.

## Data Availability

The data that support the findings of this study are available from the corresponding author upon reasonable request.
